# Incontinentia Pigmenti and Bipolar Aphthosis: An Unusual Combination

**DOI:** 10.5402/2011/814186

**Published:** 2011-03-07

**Authors:** G. Márquez Balbás, M. A. González-Enseñat, A. Vicente, L. Creus-Vila, J. Antón, P. Umbert-Millet

**Affiliations:** ^1^Servicio Dermatología, Hospital Universitari Sagrat Cor, 08029 Barcelona, Spain; ^2^Servicio Dermatología, Hospital Sant Joan de Déu Barcelona, 08950 Barcelona, Spain; ^3^Servicio Reumatología, Hospital Sant Joan de Déu Barcelona, 08950 Barcelona, Spain

## Abstract

Incontinentia pigmenti (IP) is an uncommon X-linked dominant multisystem disorder, lethal in the majority of affected males in utero and variably expressed in females. The cutaneous manifestations are diagnostic and classically occur in four stages: vesicular, verrucous, hyperpigmented, and atrophic. The skin lesions are typically spread along the lines of Blaschko, and they are usually present at birth. It may be variably accompanied by dental, ocular, neurologic, bones and joints, and development anomalies. The genes IP has been mapped to Xq28. Mutations in the NEMO/IKK*γ* gene, located at Xq28, have been found to cause expression of the disease. Behçets disease is a multisystem disorder consisting of recurrent oral aphtae, genital ulcers, pustular skin eruption, and uveitis. Occasionally there are other articular, neurological, intestinal, or vascular abnormalities. This disease is rare in children. Here, we report a case of a 16-year-old female with the rare combination of incontinentia pigmenti and an aphthosis bipolar, and we discuss the probably relationship between these two diseases.

## 1. Case Report

**Figure 1:**
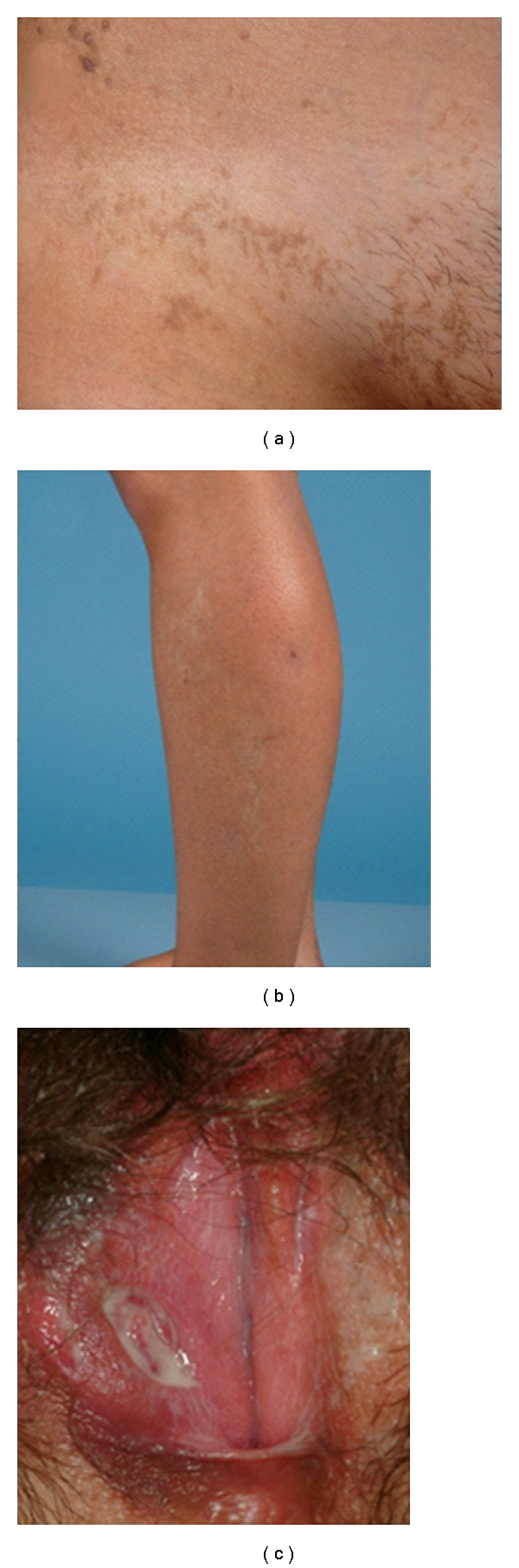
(a, b) This figure show the classical reticulated pigmented lesions in pubis and leg, typically spread along the lines of Blaschko, of Incontinentia Pigmenti. (c) shows a deep and well-defined genital ulcer, in the right labia majora.

A 16-year-old female consulted for a reticulated pigmentation in skin (Figures 1(a) and 1(b)). Clinical examination showed brownish hyperpigmented lesions in a linear pattern, following Blaschko lines, in extremities and trunk. She has been diagnosticated at first month of life of IP, for her cutaneous lesions with vesiculo-bullous at birth, followed by verrucous lesions, and the histologic findings from the skin biopsy which were compatible with IP. 

Since the age of 13, she had recurrent oral aphtae on the tongue and lips. She also developed genital ulcers, which were deep, well-defined, and clean (Figure 1(c)). These lesions were very painful, and they were resistant to topical corticosteroids, oral antibiotics, and oral antivirals. The biopsy taken from the genital ulcer was nonspecific, and it showed some fibrin debris and absence of herpetic inclusions. Clinical examination did not showed any other cutaneous lesions.

The cardiac and ophthalmologic revision showed no alteration. The patient has been diagnosticated of a cerebellopontine angle arachnoid cyst when she was 8 years old. She was asymptomatic, and the encephalogram test was strictly normal. Psychomotor development was appropriated for her age. No immunological dysfunction has been observed during the evolution of our patient.

The blood analysis showed no abnormalities, including tests for HIV, Epstein-Barr virus, cytomegalovirus, *Treponema pallidum*, chlamydya, IgG, IgA, IgM, antitransglutaminase antibody, antiendomisium antibody, ANA, anti-DNA, anti-Ro, anti-La, anti-Sm, and rheumatoid factor. The HLA0-B27 and HLA-B51 were also negative. The intradermal test with tuberculin was also negative.

The molecular genetic studies from the patient and her mother did not confirm the diagnosis of IP, as it showed no genomic deletion between exons 4 and 10 for the NEMO gene. 

The patient was treated with oral prednisone, tapering doses for several weeks. Then, we added colchicine as a long-term treatment. When recurrences occured, we added oral prednisone for several days.

## 2. Discussion

Several cases of IP with immune defects have been reported [1–3]. Some investigators [4] think that the clinical picture of IP is the result of an autoimmune reaction against ectodermic cellular clones that develop during fetal life and have surface antigens that are abnormal, because of a mutated X chromosome. The lack of immune tolerance is explained as due to a delayed expression of the modified antigens or to the appearance of a “prohibited” antigen on the surface of the ectodermic cells.

The deletion or larger frameshift mutations that completely impair NEMO function result in IP, a X-linked dominant condition in women and a lethal condition in male fetuses. The deletion between exons 4 and 10 is present in 80 percent of the patients, but a negative result does not exclude the presence of other mutations of the gene NEMO. NEMO is a scaffold protein which is associated with several genetic diseases that exhibit primary immunodeficiency, as the anhidrotic ectodermal dysplasia [5]. The transcription factor NF-*κ*B plays an important role in the immune response [5], and it has been associated with several genetic diseases that exhibit primary immunodeficiency. NEMO is necessary to activate NF-*κ*B and induces gene transcription that induces inflammatory and immune responses.

There are many data in the literature demonstrating involvement of the immune system in Behçet's disease: the patergy, the high levels of IgM, IgG and IgA, high titers of circulating immune complexes, high serum levels of C_9_ [6, 7], and increased leukocyte chemotaxis, even though investigators are not certain whether this reflects a leukocyte or a serum abnormality [7, 8]. In some other cases, however, decreased chemotaxis has been found [9]. The diagnosis of Behçet's disease is based on clinical criteria, because of the absence of a pathognomonic laboratory test. The diagnosis is made in all patients with one major criteriou (oral aphtae) and two minor criteria (genital ulcers, positive pathergy test, ocular or cutaneous involvement). The period between the appearance of an initial symptom and a major or minor secondary manifestation can be up to a decade in many cases. However, the disease may be suspected, although not meeting the criteria, according to the progress of each patient. 

The transcription factor NF-*κ*B plays an important role in the immune response [5], and it has been associated with several genetic diseases that exhibit primary immunodeficiency. NEMO is necessary to activate NF-*κ*B and induces gene transcription that induces inflammatory and immune responses.

To our knowledge, there are four cases reported with the combination of IP and Behçet's disease [9–12]. All the cases previously reported had aphthosis bipolar, as our patient. The coexistence of the two syndromes appears to be a chance occurrence. There have been no studies of histocompatibility antigens in IP indicating that it is related to Behçet's disease [10]. Some authors have reported that there may be common immunological abnormalities between the two syndromes. The presence of an impaired leukocyte chemotaxis in several patients with both syndromes seems to suggest a common pathogenesis basis. In our patient, Behçet's disease was suspected for the recurrent oral aphtae and genital ulcers and the description of a probable relationship with IP.

We thought it is important to bring a new case of the association of a probable Behçet's disease and IP, as there are only four cases reported. We consider it is important to monitor the patient in the future, for the possibility of developing Behçet's disease, which has been described in association with IP in the literature.
